# Blood Relatives: Splicing Mechanisms underlying Erythropoiesis in Health and Disease

**DOI:** 10.12688/f1000research.15442.1

**Published:** 2018-08-30

**Authors:** Kirsten A. Reimer, Karla M. Neugebauer

**Affiliations:** 1 Molecular Biophysics and Biochemistry, Yale University, New Haven, Connecticut, 06520, USA

**Keywords:** erythropoiesis, ß-thalassemia, myelodysplastic syndrome (MDS), globin, pre-mRNA splicing, spliceosome, nonsense-mediated decay (NMD), intron retention, RNA-seq, CRISPR/Cas

## Abstract

During erythropoiesis, hematopoietic stem and progenitor cells transition to erythroblasts en route to terminal differentiation into enucleated red blood cells. Transcriptome-wide changes underlie distinct morphological and functional characteristics at each cell division during this process. Many studies of gene expression have historically been carried out in erythroblasts, and the biogenesis of β-globin mRNA—the most highly expressed transcript in erythroblasts—was the focus of many seminal studies on the mechanisms of pre-mRNA splicing. We now understand that pre-mRNA splicing plays an important role in shaping the transcriptome of developing erythroblasts. Recent advances have provided insight into the role of alternative splicing and intron retention as important regulatory mechanisms of erythropoiesis. However, dysregulation of splicing during erythropoiesis is also a cause of several hematological diseases, including β-thalassemia and myelodysplastic syndromes. With a growing understanding of the role that splicing plays in these diseases, we are well poised to develop gene-editing treatments. In this review, we focus on changes in the developing erythroblast transcriptome caused by alternative splicing, the molecular basis of splicing-related blood diseases, and therapeutic advances in disease treatment using CRISPR/Cas9 gene editing.

## Introduction

**Figure 1.**
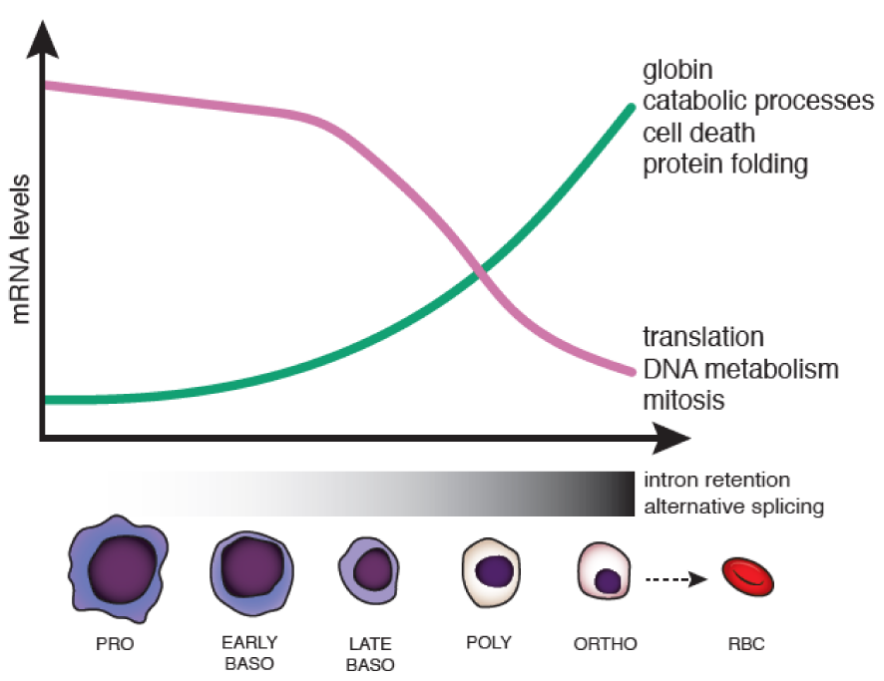
Changes in gene expression and splicing occur during terminal erythroid differentiation. Erythropoiesis is characterized by changes in cell morphology, including nuclear size, color (due to hemoglobinization), and chromatin condensation, which are coordinated with changes in gene expression. During terminal erythroid differentiation, cells progress from proerythroblasts (PRO), to basophilic erythroblasts (EARLY and LATE BASO), to polychromatophilic erythroblasts (POLY), to orthrochromatic erythroblasts (ORTHO), before enucleation to become red blood cells (RBCs) (also called reticulocytes). In human erythroblasts, a subset of genes is downregulated—some top associated Gene Ontology terms are shown to the right (purple line)—while a subset of genes is concurrently upregulated (green line). Changes in splicing occur in the later stages of erythropoiesis (mostly from late baso to ortho), including increased alternative splice site usage and intron retention.

Erythropoiesis is the developmental pathway by which red blood cells (RBCs)—specialized hemoglobin-containing cells that deliver oxygen throughout the body—are produced from hematopoietic stem and progenitor cells (HSPCs). Morphologically, this includes the loss of the cell nucleus and acquisition of a characteristic disk-like shape ( Figure 1). Early molecular biologists identified erythropoiesis-associated gene expression patterns such that the globin genes are among the best-understood eukaryotic genes. β-globin was among the first proteins to be sequenced and was the first protein to be characterized structurally by using X-ray crystallography. The β-globin gene and mRNA were also among the first to be cloned. These advances facilitated early discoveries in gene regulation, such as the transcriptional control of globin genes by long-range enhancer and repressor elements present in the locus control region ^1^. This system of transcriptional regulation is currently being exploited to discover how chromosomal regions interact and how chromatin looping might be a therapeutic target in diseases of RBCs ^2, 3^.

RNA biology is an area in which erythropoiesis, globin gene regulation, and disease mutations have led to fundamental discoveries. The globin genes contain two non-coding intervening sequences (introns), which are removed by the spliceosome in a process termed pre-mRNA splicing ^4^. Globin pre-mRNA was an early model substrate for the investigation of splicing mechanisms ^5, 6^, and mutations in globin genes at the 5′ and 3′ boundaries of the introns—termed splice sites (5′ and 3′ SSs)—proved to be the cause of various forms of thalassemia ^7^. Thalassemias are hemoglobin deficiencies resulting from aberrant globin expression. Some thalassemia mutations cause intron retention (IR) and lead to nonsense-mediated decay (NMD), a major gene regulatory mechanism that degrades mRNA transcripts containing premature termination codons (PTCs) present in retained introns ^8, 9^. Finally, other thalassemia mutations disrupt the nucleotide sequence that signals 3′ end cleavage and polyadenylation of β-globin mRNA, showing the importance of this RNA processing mechanism in health and disease. This review will focus on the common roles of splicing in diseases of erythropoiesis with an emphasis on recent insights into the transcriptomes of developing RBCs, the effects of splicing factor mutations that drive myelodysplastic syndromes (MDSs), and current efforts to restore normal globin expression in thalassemias using CRISPR/Cas-mediated genome editing.

## Transcriptome-wide changes during erythropoiesis

During erythropoiesis, each cell division is coincident with major changes in gene expression, resulting in daughter cells that are morphologically and transcriptionally distinct from the mother cell ^10^. Transcriptome-wide profiling using RNA sequencing (RNA-seq) has allowed unbiased dissection of changes that occur along this developmental pathway ^11^. The greatest number of changes in gene expression in human erythroblasts—either upregulation or downregulation—occurred between the late basophilic to polychromatic and polychromatic to orthochromatic stages, where roughly equal numbers of genes are upregulated as are downregulated. These transcripts were enriched for differently annotated functions reflecting cellular events in the differentiation process ( Figure 1), emphasizing the changing transcriptional landscape that underlies massive globin gene expression in terminal stages of erythroid development. In contrast, when mouse erythroblasts were analyzed in the same manner, the overwhelming majority of genes, including key transcription factors, were downregulated. The cause of species-specific differences in transcriptome changes is not immediately clear but likely reflects distinct properties of human and mouse erythrocytes, including differences in size, life span, oxygen-carrying capacity, and metabolism ^10^. An analysis of chromatin accessibility, histone modifications, and transcription factor binding in a mouse embryonic stem cell model of hematopoiesis has revealed a complex regulatory network that drives changes in the transcriptome during differentiation ^12^. It remains to be seen whether these mechanisms differ between human and mouse erythroblasts to explain the pronounced differences in transcriptomes.

## Splicing regulation in normal erythropoiesis

Splicing can contribute to the regulation of transcript levels by activating cellular programs, such as NMD, to reduce transcript levels. Moreover, alternative splicing leads to the expression of different transcripts and protein products from the same gene ^4^. How does splicing regulation contribute to transcriptome diversity in erythroid development? Early work using microarrays to detect changes in splicing during erythropoiesis found altered splicing in known trans-acting splicing factors (for example, SNRP70, HRNPLL, and MBNL2), which are RNA-binding proteins that regulate how the spliceosome assembles on pre-mRNA and how different 5′ and 3′ SSs are chosen ^13^. This suggested a regulatory feedback loop, whereby changes in splicing factors could affect the splicing of many downstream genes necessary for development. Subsequent work has focused on identifying stage-specific changes in splicing transcriptome-wide using RNA-seq, a less biased approach that does not rely on known intron–exon boundaries ^10^. In addition, mapping the gene expression networks governed by splicing in erythroid differentiation has aided in identifying the functional significance of splicing regulation ^14^.

One of the first and most well-characterized examples of alternative splicing in erythropoiesis is the stage-specific inclusion of exon 16 of the *4.1R* gene, which encodes a protein that is crucial for erythrocyte membrane integrity ^13, 15^. Changes in expression levels and specific binding of the hnRNP A/B protein affect this developmental switch ^15^. Since then, alternative splicing has emerged as a more widespread phenomenon ^16– 18^. The muscleblind-like protein 1 (MBNL1) is a sequence-specific splicing factor that undergoes extensive alternative splicing during differentiation ^17^. Cheng *et al*. showed that a specific Mbnl1 isoform which includes the alternative exon 5 accumulates in the nucleus in later stages of erythroid differentiation ^17^. The inclusion Mbnl1 isoform is responsible for regulating the splicing of downstream genes important for erythroid differentiation, as knockdown of the Mbnl1 inclusion isoform alone blocked differentiation and caused defects in proliferation. Mirroring the previous findings observed by microarrays, Pimentel *et al*. report a program of highly dynamic alternative isoform switching in late-stage human erythroblasts using RNA-seq ^19^. An increase in steady-state levels of transcripts containing PTCs, which likely trigger NMD of these transcripts, was observed in the later stages of differentiation, suggesting that alternative splicing coupled to NMD may be a novel, stage-specific gene regulatory mechanism.

## Intron retention is a regulatory mechanism during hematopoiesis

IR is a class of alternative splicing wherein an intron is not removed by the spliceosome, potentially introducing PTCs and targeting the transcript for NMD. Alternatively, it is possible that certain intron-retained transcripts remain in the nucleus and undergo splicing with delayed kinetics ^20– 23^. IR was only recently recognized as a widespread occurrence ^24, 25^, and developing erythroid cells exhibit robust IR. Pimentel *et al*. showed that late human erythroblasts accumulate hundreds of transcripts containing retained introns and that these IR transcripts are enriched for splicing factors and iron-homeostasis factors ^26^. These results were corroborated at the single-cell level in human immortalized myelogenous leukemia K562 cells ^27^. The top three categories of nuclear IR transcripts by gene ontology analysis were RNA metabolism, RNA splicing, and the C complex spliceosome. The retained introns detected in late human erythroblasts were more likely to be found next to alternative exons that contained PTCs ^26^, in line with previous studies suggesting that IR followed by NMD is an important mechanism that regulates levels of splicing factors ^28^.

How is IR triggered during erythropoiesis? Key insights are emerging from studies of transcripts encoding the important core splicing factor SF3B1. SF3B1 expression is also subject to IR during erythroid differentiation suggesting that SF3B1 regulation by IR may constitute a regulatory hub leading to the downregulation of transcripts encoding other splicing factors ^26^. Indeed, a series of highly conserved cryptic SSs were identified for their activity in promoting IR in SF3B1 transcripts ^29^. The identified intronic sequences are sufficient to promote IR in SF3B1 and can also promote retention when inserted into other introns. The cryptic exons generated by these SSs are proposed to act as splicing decoys, sequestering components of the spliceosome and ultimately preventing productive splicing by blocking the appropriate cross-intron interactions needed to define the intron for splicing. Alternatively, reduced levels of SF3B1 might preferentially affect splicing efficiencies or the half-lives of (pre-)mRNAs encoding splicing factors or both. Although we presume that most of these instances reflect the downregulation of IR transcripts, the possibility that certain splicing events are delayed remains. Interestingly, delaying gene expression through IR is physiologically relevant in other cell types, including developing spermatocytes, neuronal cells, platelets, granulocytes, and stimulated macrophages ^24, 30– 33^. In the case of erythroid differentiation, how introns are retained in a seemingly stage-specific and cell type-specific way remains to be fully understood.

## Misregulation of splicing in diseases of erythropoiesis

Misregulation of splicing underlies a growing number of human diseases ^34– 37^. Generally, mutations in either cis or trans can affect splicing outcomes. Cis mutations may disrupt the intrinsic sequences that demarcate SSs in a transcript (5′ and 3′ SSs). In contrast, mutations in any number of the core spliceosome machinery can produce splicing defects in trans, which causes deleterious effects for a large number of downstream splicing substrates. Both types of splicing defects have been characterized in the erythroid lineage ^38^.

## β-thalassemia

**Figure 2.**
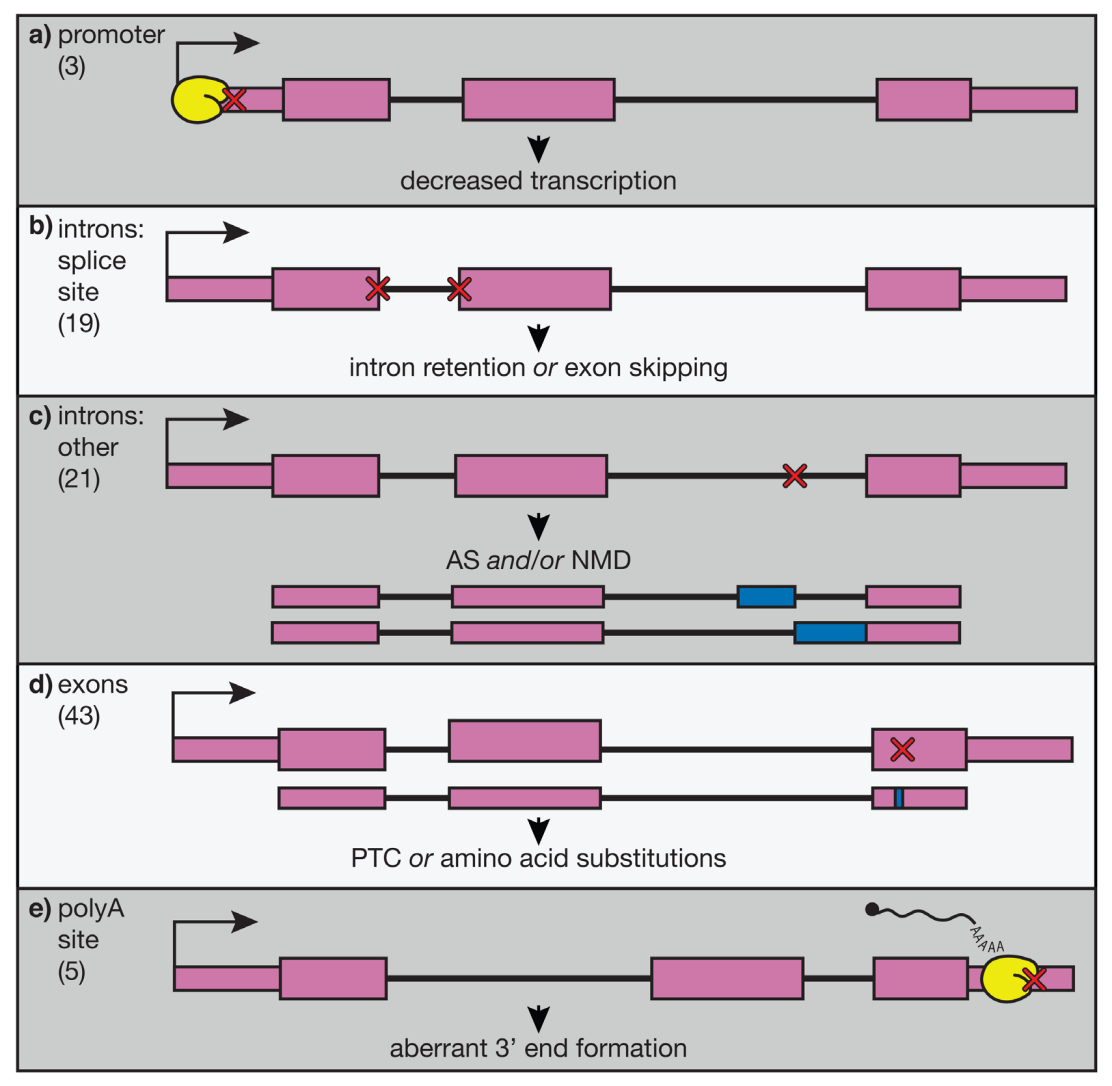
Single-nucleotide mutations in key regulatory regions of the β-globin gene disrupt expression in β-thalassemia. Point mutations in varying regions of the β-globin gene are shown schematically, and the frequencies of these mutations listed in the HbVar database ( http://globin.bx.psu.edu/hbvar/menu.html) are shown in brackets at the left. RNA polymerase is shown in yellow. Gene regions are divided into **a**) promoter, **b**) splice sites, **c**) other intronic regions, **d**) exons, and **e**) polyadenylation site. These mutations (red X’s) have varying effects, illustrated below each example, but all lead to decreased or abolished expression of the β-globin transcript. AS, alternative splicing; NMD, nonsense-mediated decay; PTC, premature termination codon.

β-thalassemias are a family of disorders defined by mutations in the β-globin gene, causing a reduction of β-globin mRNA, insufficient hemoglobinization of maturing RBCs, and anemia ^39^. β-thalassemia is one of the most prevalent diseases caused by somatic mutations worldwide, yet currently the only available curative treatment is an allogenic transplant of HSPCs from a matched donor. This option is unavailable for many patients because of the cost of treatment and limited availability of matched donors. Although we possess a quite-thorough understanding of the molecular basis and pathophysiology of this disease, better treatments are still sorely needed. Many β-thalassemia patients are dependent on transfusions from blood donors to maintain proper levels of healthy, circulating RBCs. However, this therapy often leads to complications related to iron overload, including organ damage. The majority of β-globin alleles that cause thalassemia contain point mutations ( Figure 2). These mutations can affect virtually any step in the correct expression of β-globin mRNA from transcription initiation ( Figure 2A), to splicing ( Figure 2B, C), to 3′ end cleavage and polyadenylation ( Figure 2E). Because of this, β-thalassemia is an attractive target for applying genome-editing tools to correct β-globin mRNA processing and expression, providing a potential cure for β-thalassemia.

Several methods attempting to restore β-globin mRNA expression in β-thalassemia and sickle cell disease patients have been reported ^40^. One method that has provided some success in allowing patients to become transfusion independent is using a lentiviral vector to integrate a wild-type copy of the β-globin gene randomly into the genome. However, random integration of a viral vector comes with the risk of disrupting other parts of the genome. Using CRISPR/Cas9, researchers have been able to specifically edit a point mutation in the β-globin gene in human HSPCs to restore the wild-type sequence ^41^. Importantly, they were also able to specifically enrich for edited cells. Enrichment is a hurdle for these types of genome-editing approaches, since edited HSPCs must be expanded *ex vivo* before being transfused back into the patient. Similar progress has been made using CRISPR/Cas9 to edit HSPCs to correct the single nucleotide that causes sickle cell disease ^42^. Alternatively, some genome-editing approaches aim to edit distal regulatory regions to increase the expression of fetal hemoglobin derived from the γ-globin gene, which is normally turned off just after birth; when expressed in adult cells, γ-globin can compensate for a lack of functional β-globin ^40, 43, 44^. With advancements in editing efficacy and *ex vivo* expansion, gene editing promises to soon be a feasible treatment for β-thalassemia and other genetically encoded diseases of the blood.

## Myelodysplastic syndromes

**Figure 3.**
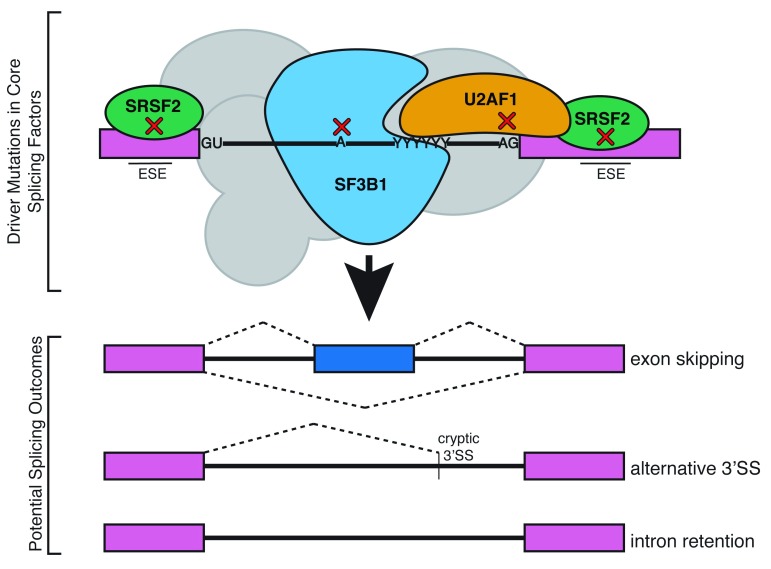
Driver mutations in myelodysplastic syndromes introduce single amino acid changes to core splicing factors. Somatic mutations in general splicing factors U2AF1, SRSF2, and SF3B1 have been implicated in myelodysplastic syndromes. Although these proteins are involved in the recognition of splice site sequences in all pre-mRNAs, tissue-specific splicing defects are observed in blood cells. These mutations lead to aberrant splicing in erythroblasts in a wide range of transcripts. ESE, exonic splicing enhancer; SS, splice site.

MDSs are characterized by ineffective erythropoiesis and a predisposition to develop acute myeloid leukemia despite broad phenotypic heterogeneity ^45– 47^. Recently, recurrent mutations associated with MDS have been reported in core spliceosomal proteins ^48– 52^. While these proteins share a role in normal pre-mRNA splicing, these MDS mutant alleles can produce varied outcomes in aberrant splicing. It is puzzling to imagine how mutations in core splicing factors, which carry out splicing in every tissue in the body, drive malignancy specifically in the blood lineage ^53, 54^. Additionally, it is unknown how these neomorphic alleles provide an advantage for the mutant cells in order to drive leukemogenesis. MDS alleles are in all cases mutually exclusive between splicing factors, and the affected tissue is always heterozygous ^35, 48^. The most frequently occurring splicing factor mutations associated with MDS are found in the proteins SF3B1, U2AF, and SRSF2 ( Figure 3), and mutations are found less commonly in other splicing factors (ZRSR2, U2ASF65, PRP40B, and SF1) ^48^. The MDS alleles cause non-overlapping patterns of alternative splicing, suggesting that a complex mechanism of aberrant splicing promotes MDS or else that disease progression is promoted by alternative activities of these RNA-binding proteins unrelated to splicing ^55^.

## SF3B1

SF3B1 is an essential component of the U2 snRNP that binds the intron branch point sequence (BPS) during spliceosome assembly and helps to identify SSs that are used in catalysis. SF3B1 mutations are generally associated with disease, and the most common point mutant found in subtypes of MDS is *SF3B1 K700E* ^49, 56– 59^. Mutations found in MDS promote the recognition of non-consensus BPS and activate cryptic 3′ SSs ^60– 62^; however, the mechanism for how altered splice selection translates to hematological malignancy is still under debate. In CD34 ^+^ cells from patients with SF3B1 mutations, genes involved in cell cycle regulation, iron homeostasis, DNA damage, and RNA processing are deregulated ^63^. In order to explain the link between aberrant splicing and gene expression, two studies examined RNA-seq data from mutant SF3B1 patient samples ^64, 65^. The most common aberrant splicing event was use of a cryptic 3′ SS, via recognition of an alternate BPS upstream of the canonical BPS, and about half of the affected transcripts were targets for NMD ^64^. In *Sf3b1 K700E* knock-in mice, alternate 3′ SS usage was observed, as was inefficient hematopoiesis ^66, 67^. However, these mice did not develop other MDS phenotypes such as ringed sideroblasts as seen in humans, and this was potentially due to differences in disease mechanisms between human and mouse. Additionally, aberrantly spliced transcripts in these mouse models showed little overlap with changes observed in human patients, perhaps owing to the decreased conservation of intron sequences between species. Several investigations have also looked into the role of SF3B1 outside of splicing. SF3B1 was found to associate with mononucleosomes in HeLa cells, preferentially over exons, and SF3B1 association with chromatin influenced splicing ^68^. However, recent work by Murthy *et al*. also found an association of SF3B1 with exons in chromatin, but this association did not predict splicing outcomes ^69^. These results point to the role of SF3B1 as a chromatin modifier.

## SRSF2

SRSF2 is a member of the serine/arginine (SR)-rich family of proteins, which are involved in regulating SS selection for both constitutive and alternative splicing by binding exonic sequence enhancer. Multiple lines of evidence support a role for mutant SRSF2 in driving myelodysplasias by promoting changes in alternative splicing. In a K562 cell line expressing the most common P95H mutation, alternative splicing events were correlated directly with the altered ability of the P95H mutant to bind a specific RNA motif ^70^. SRSF2 is essential for hematopoiesis, and *Srsf2* gene knockout in mice is embryonic lethal ^71^. In one study, the P95H mutant was found to disrupt the recognition of exonic splicing enhancer regions, and in the case of the oncogene *Ezh2*, this resulted in the inclusion of a toxic exon and a decrease in *Ezh2* transcript levels ^72^. Similarly, the P95H mutant expressed in an MDS cell line was shown to specifically affect alternative splicing of genes that are important for cancer development and apoptosis ^71^. These results were generally corroborated with a conditional knock-in mouse model to look at steady-state effects rather than in the regenerative context of lethally irradiated mice as in previous mouse models ^73^. Kon *et al*. found increased occurrences of alternative splicing in about 20% of transcripts ^73^. However, in these mice, there were only a few differentially spliced genes that were targets for NMD, and no change in the expression of *Ezh2*, suggesting that the context of regenerative stress may have an impact on the role of SRSF2 in promoting aberrant alternative splicing. Using a technique that measure protein–RNA interactions genome-wide, SRSF2 P95H was found to differentially bind alternative exons, but interestingly this does not predict the outcome of alternative splicing ^74^. The genes most affected were RNA processing factors, suggesting a cascade effect, where mutant SRSF2 promotes misregulation of splicing factors and thereby causes aberrant splicing and downregulation of a host of downstream targets.

## U2AF1

U2AF1 (also known as U2AF35) binds to most 3′SSs, assisting the assembling spliceosome in identification of the 3′SS to be used during splicing catalysis. Unbiased whole-genome sequencing found the S34F mutation in around 9% of 150 patients with *de novo* MDS, and this mutation promoted exon skipping in a minigene reporter ^75^. RNA-seq data from patients with this same mutation showed again a small increase in exon skipping, and the nucleotide just upstream of the 3′ SS was a predictor of exon usage for mutant but not wild-type splicing ^76^. These results were verified by using RNA-seq data from 167 patients with acute myeloid leukemia, confirming that U2AF1 S34F/Y mutants show a preference for CAG over UAG 3′ SSs, causing downstream aberrant splicing events ^77^. However, in both of these cases, the downstream targets with altered splicing were variable and did not contain much overlap.

To investigate the effects of expressing the S34F mutant of U2AF1 in an animal model, Shirai *et al.* developed a doxycycline-inducible transgenic mouse carrying the allele in the myeloid, lymphoid, stem, and progenitor cell lineages ^78^. After transplant of the transgenic bone marrow to lethally irradiated mice, peripheral blood total white blood cell counts were decreased, as was the case for B cells and monocytes ^78^. However, there was no change in RBC or platelet counts, and the mice showed no evidence of bone marrow dysplasia and did not develop MDS or acute myeloid leukemia after one year of transgenic *U2af1 S34F* expression. By analyzing transcriptome sequences of myeloid progenitors from the induced transgenic mice, they identified 742 cases of alternate splicing junctions, and these were mostly alternate 3′ SS usage with an enrichment for CAG 3′ SS sequence over UAG in wild-type controls. Finally, in an attempt to link altered splicing patterns to the cause of myeloid disease, Park *et al*. derived cell lines with the S34F mutation and showed that, in addition to aberrant alternative splicing, many transcripts are alternately processed at the 3′ end, specifically through use of a distal cleavage and polyadenylation site ^79^. They show that one such alternatively processed transcript, *Atg7*, causes an autophagy defect that may explain how changes in splicing and RNA processing can drive oncogenic transformation. MDS patients harboring the S34F mutation had increased levels of *Atg7* mRNA with the distal cleavage and polyadenylation site. Importantly, the authors show that use of the distal site results in translation repression and that the corresponding decrease in ATG7 protein levels is sufficient to transform cells.

The recent discovery of splicing factors as commonly mutated proteins in MDS was unexpected. Although these mutations affect proteins in a common pathway, the outcome in each case is a distinct pattern of altered splicing. Additionally, these mutations are associated with variable prognoses and subclasses of MDS. SF3B1 is strongly associated with a ringed sideroblast phenotype and a relatively positive prognosis, whereas U2AF1 and SRSF2 are associated with more advanced myelomonocytic leukemia and with worse outcomes ^52^. One unifying mechanism proposed by Chen *et al.* is that while mutations in splicing factors cause distinct splicing defects, they cause a common replication stress by triggering elevated R-loop formation ^80^. The authors show that increased R-loops compromise hematopoiesis and that resolution of R-loops by overexpression of RNase H partially rescues proliferation. This is an intriguing mechanism by which splicing factors may play auxiliary roles in transcriptional regulation. They propose that this insult to genomic stability may be a common mechanism which makes progression to more severe disease phenotypes more likely when other mutations are coexisting. Retained introns have been proposed to act as substrates for R-loop formation, contributing to genomic instability and DNA damage in the case of severe spinal muscular atrophy ^81^. More work will need to be done to understand the contribution of both the splicing and the genomic stability defects of mutant splicing factor alleles on MDS.

Mutations in several other RNA-binding proteins have been proposed to contribute to misregulation of splicing during erythropoiesis. For example, RBM38 has been shown to regulate alternative splicing in terminal erythropoiesis, specifically activating inclusion of exon 16 of the *4.1R* transcript ^82^, a signature alternative splicing event in RBC development (see above). Naturally occurring human variants in RBM38 expression were discovered through a high-throughput genome-wide association study screen and validated to show changes in a subset of genes that are alternatively spliced during terminal erythropoiesis ^83^. Interestingly, RBM38 was additionally shown to interact with eIF4G in the cytoplasm to enhance the translation of a subset of mRNAs in terminal differentiation ^84^. Moreover, an alternatively spliced isoform of the transcriptional repressor GFI1B produced by a natural variant causes defects in megakaryopoiesis but not erythropoiesis, suggesting that variation among individuals can play a role in developmental dynamics ^85^. Future investigations should illuminate further connections among alternative splicing, transcription, and translation during hematopoiesis.

## Conclusions

Erythropoiesis provides an excellent model in which to study RNA splicing in both healthy and diseased states. Recent work identifying alternative splicing ^19^ and IR ^26, 29^ as major regulatory mechanisms associated with differentiation will no doubt yield insight into the complexity of how tissue-specific changes in splicing are relayed to affect gene expression. Mutations affecting core spliceosomal proteins (SF3B1, U2AF1, and SRSF2) as well as genes important for mature RBC function (for example, β-globin) have revealed aberrant splicing which leads to hindered erythropoiesis, often in unexpected ways. Promising treatments for MDS, in particular, rely on modulating splicing as a mechanism to specifically target defective splicing in the myeloid lineage ^86– 88^. Many questions remain as to how mutations in ubiquitous splicing factors specifically cause defects in the myeloid lineage and what the molecular mechanism is for how downstream targets of these alternatively spliced transcripts contribute to disease progression.

## Abbreviations

BPS, branch point sequence; HSPC, hematopoietic stem and progenitor cell; IR, intron retention; MBNL1, muscleblind-like protein 1; MDS, myelodysplastic syndrome; NMD, nonsense-mediated decay; PTC, premature termination codon; RBC, red blood cell; RNA-seq, RNA sequencing; SS, splice site
